# USF1 regulated circPRDM4 modulates tumorigenesis and immune escape in chemoresistant cervical cancer

**DOI:** 10.1111/jcmm.17945

**Published:** 2023-09-04

**Authors:** Yan Zhang, Xing Li, Jun Zhang, Lin Mao, Zou Wen, Mingliang Cao, Xuefeng Mu

**Affiliations:** ^1^ Department of Obstetrics and Gynecology Renmin Hospital of Wuhan University Wuhan China

**Keywords:** cervical cancer, circPRDM4, immune escape, USF1

## Abstract

Cervical cancer (CC) represents a major global health concern, characterized by chemoresistance and immune evasion mechanisms. Circular RNAs (circRNAs), which play a crucial role in cancer pathogenesis, particularly in the case of CC, have gained significant attention. The primary objective of this study was to investigate the functional significance of circRNAs in chemoresistant CC. A significant upregulation of circPRDM4 expression in chemoresistant CC cells. To investigate the functional consequences, we conducted circPRDM4 knockdown experiments, which resulted in the effective blockade of immune escape mechanisms employed by chemoresistant CC cells. Furthermore, circPRDM4 knockdown demonstrated a significant suppression of tumorigenesis in CC cells, highlighting its contribution to the oncogenic potential of CC. Investigating the regulatory mechanisms involved, we found that the transcriptional factor upstream stimulatory factor 1 (USF1) acts as an inducer of circPRDM4 expression. Remarkably, USF1 was found to effectively modulate CC cell immune escape via its interaction with circPRDM4. Moreover, our results revealed that USF1 is intricately involved in CC cell tumorigenesis through the regulation of circPRDM4. Collectively, our study elucidates the significant roles of circPRDM4 and its upstream regulator USF1 in chemoresistant CC cells. These findings underscore the importance of circRNAs in CC pathogenesis and provide valuable insights into the mechanisms underlying immune escape and tumorigenesis.

## 1 INTRODUCTION

Cervical cancer (CC) remains a distressing global reality, persistently holding its place as the fourth leading cause of cancer‐related fatalities among women across the world.^1^ Despite remarkable advancements in preventive measures and treatment approaches over recent decades, the outlook remains bleak for patients grappling with advanced or recurring CC.^2^ In the context of patients grappling with substantial localized lesions or systemic metastases, chemotherapy assumes a position of utmost significance.^3^ It holds the potential to enhance prognosis, attenuate localized lesions, facilitate subsequent treatment interventions and impede the progression of distant metastases.^4^ As a first‐line agent for CC chemotherapy, cisplatin (DDP) currently plays a prominent role. However, the curative impact of this treatment is restricted due to the emergence of chemo‐resistance in a significant subset of patients.^5^ Hence, the identification of new prognostic biomarkers assumes significance as it would furnish clinicians with potential therapeutic targets for the implementation of personalized treatment regimens.

Circular RNAs (circRNAs) are a distinct class of RNA molecules that arise from precursor‐mRNAs through a process called back‐splicing. Unlike linear RNAs, circRNAs possess a covalently closed loop structure without a specific 5′‐3′ polarity or a polyadenylated tail. While the complete understanding of circRNAs' functionality remains elusive, recent investigations have shed light on their diverse roles, including acting as sponges for proteins or miRNAs, encoding polypeptides or forming stable RNA‐protein complexes to orchestrate downstream biological processes.^6^, ^7^, ^8^, ^9^ The importance of circRNAs in various diseases, particularly cancer has gained significant attention in recent studies.^10^, ^11^, ^12^ Extensive research has unveiled the critical pathological roles played by circRNAs, establishing their close association with the development and progression of various cancers. Dysregulation of circRNAs has emerged as a significant contributing factor in cancer pathogenesis.^10^, ^13^ Chen et al.^14^ made a noteworthy discovery regarding the role of circMTO1 in the chemoresistance of CC. Their findings demonstrated that circMTO1 acts as a miRNA sponge, exacerbating the chemoresistance of CC cells.^14^ Through their study, Guo et al. provided compelling evidence regarding the significant involvement of hsa_circ_0023404 in the progression of CC. Their findings revealed that hsa_circ_0023404 plays a crucial role by reducing the sensitivity of CC cells to cisplatin treatment.^15^ The involvement of circRNAs in the development of chemoresistance in CC is of paramount importance. However, the precise underlying mechanisms by which circRNAs regulate CC chemoresistance remain poorly elucidated.

Our study aims to explore a novel functional circRNA in CC chemoresistance. First, we utilized circRNA microarray analysis on two pairs of CC chemoresistance (DDP) cell lines. It was found that circPRDM4 was found to be a most significantly upregulated gene in both DDP‐resistant CC cells. By performing a serial experiment, it was found that circPRDM4 knockdown markedly promoted the sensitivity of CC cells to cisplatin treatment and blocked immune escape of CC cells. Additionally, circPRDM4 knockdown significantly inhibited tumorigenesis of CC cells. Mechanically, upstream stimulatory factor 1 (USF1) was found to transcriptionally regulate circPRDM4 expression and modulate chemoresistance and immune escape of CC cells through inducing circPRDM4. Our findings revealed a novel USF1/circPRDM4 axis in the progression of CC cell chemoresistance and immune escape, which underscores the importance of circRNAs in CC pathogenesis and provide valuable insights into the mechanisms underlying immune escape and tumorigenesis.

## 2 MATERIALS AND METHODS

### 2.1 Cell treatment

The HeLa, SiHa, HeLa/DDP and SiHa/DDP cell lines were procured from Procell Life Science and Technology in Wuhan, China. These cell lines were cultured in Dulbecco′s modified eagle medium (DMEM) medium supplemented with 10% fetal bovine serum (FBS) and 1% penicillin–streptomycin. The cells were maintained at a temperature of 37°C in a humidified atmosphere containing 5% CO_2_. To establish drug resistance, a concentration of 500 ng/mL Dox (Sigma‐Aldrich) was added to the medium for the culture of Dox‐resistant cells. However, the Dox maintenance treatment for the resistant cells was discontinued 3 days prior to the experiment. For the experiments, the mock plasmid pcDNA3.1, small hairpin RNAs (shRNAs) targeting circRNA, and non‐specific negative control oligos (sh‐NC) were obtained from GenePharma. Lentivirus was purchased from GeneChem. The cells were seeded in six‐well plates 24 h before transfection. Transfections were carried out using Lipofectamine 3000 (Invitrogen) as per the manufacturer's instructions. The effects of knockdown or overexpression were assessed using reverse transcription quantitative polymerase chain reaction (RT‐qPCR), with RNA extraction performed 48 h after transfection.

### 2.2 CirRNA microarray analysis

Total RNA was isolated using TRIzol reagent (Life Technologies), and its concentration was determined using a NanoDrop ND‐1000 spectrophotometer. To selectively enrich circular RNA (circRNA) while removing linear RNA, Rnase R treatment (Epicentre) was performed. The enriched circRNA was then subjected to amplification and fluorescent labeling using the Super RNA Labeling kit (Arraystar), following the manufacturer's instructions. The labelled complementary RNA (cRNA) was hybridized to the Arraystar Human circRNA Array V2 (8 × 15 K). Subsequently, the slides were washed and scanned using an Agilent Scanner G2505C. The acquired images were analysed using the Agilent Feature Extraction software (version 11.0.1.1). For data processing, including quantile normalization and analysis, the limma package in R was utilized. Differential expression of circRNAs and their expression patterns were identified through fold change filtering and hierarchical clustering techniques.

### 2.3 qRT‐PCR

TRIzol reagent (Invitrogen, USA) was utilized to isolate total RNA from tissue samples or cell lines. Subsequently, the first‐strand cDNA synthesis was performed by employing a reverse transcription reagent kit from Invitrogen (USA). Real‐time PCR was conducted using the SYBR Green PCR kit (TaKaRa). Internal control genes, GAPDH and U6, were employed for normalization purposes. The relative expression levels of the target genes were determined by comparing them to the expression of GAPDH or U6 using the 2^(‐ΔΔCT) method. The real‐time PCR was carried out with the following primer sequences: circPRDM4: Forward; 5’‐GGACAGACAAGGCAGTTAACCATAT‐3′, Reverse; 5’‐GGTCACACAGAGTACACCCTGG‐3′. PRDM4: Forward; 5’‐CCGGTCGACGAAAACATGCATCACAGGATG‐3′, Reverse; 5’‐CGCGGATCCGTTATTTATGTGCAGAAAGA‐3′. USF1: Forward; 5’‐TCCCAGACTGCTCTATGGAGA‐3′, Reverse; 5’‐CGGTGGTTACTCTGCCGAAG‐3′. GAPDH: Forward; 5’‐GCATTGCCCTCAACGACCAC‐3′, Reverse; 5’‐CCACCACCCTGTTGCTGTAG‐3′. U6: Forward; 5’‐CTCGCTTCGGCAGCACA‐3′, Reverse; 5’‐AACGCTTCACGAATTTGCGT‐3′.

### 2.4 Cell Counting Kit‐8 assay

For the assessment of cell proliferation rate, a Cell Counting Kit‐8 (CCK‐8) assay was employed. The cells were seeded in 96‐well plates and cultured for 24 h until reaching a density of 3000 cells per well. Subsequently, a mixture comprising 90 μL of serum‐free medium and 10 μL of CCK‐8 reagent (Abxin) was added to each well. After incubation with the CCK‐8 mixture for 2 h, the absorbance was measured at 450 nm using a microplate reader. This measurement provided quantitative data on cell proliferation.

### 2.5 Detection of peripheral blood mononuclear cell proliferation

Peripheral blood mononuclear cells (PBMCs) were subjected to a series of steps for immunostaining. First, the cells were fixed using 4% paraformaldehyde, followed by permeabilization with 0.1% Triton X‐100 and subsequent blocking with 3% bovine serum albumin. For Ki‐67 staining, the cells were incubated overnight at 4°C with the primary antibody, anti‐Ki‐67 (ab15580, Abcam) and then with the secondary antibody, IgG (Alexa Fluor 647) (ab150075, Abcam). To visualize the nuclei, 4′,6‐diamidino‐2‐phenylindole (Sigma‐Aldrich) was used for counterstaining. After applying a sealing solution supplemented with an antifluorescence quenching agent, the slides were captured and analysed using a fluorescence microscope (Olympus) and ImageJ software.

### 2.6 Enzyme‐linked immunosorbent assay

To measure the levels of interferon‐gamma (IFN‐γ) (ab174443), tumour necrosis factor (TNF)‐α, transforming growth factor (TGF)‐β1 (ab100647) and interleukin (IL)‐10 (ab185986) in the supernatant of co‐cultured PBMCs, commercially available enzyme‐linked immunosorbent assay (ELISA) kits from Abcam were utilized. The measurements were performed according to the instructions provided by the manufacturer.

### 2.7 Western blotting

About 50 μg of total proteins was loaded onto an SDS–PAGE gel and transferred into a PVDF membrane. The membrane was blocked using 5% non‐fat milk and incubated overnight at 4°C with primary antibodies. The next day, the membrane was incubated with secondary antibodies. Following that, the membrane was exposed to an ECL solution and imaged to detect the protein bands. The antibodies used in this study were anti‐IgG (Abcam), anti‐USF1 (Abcam), and anti‐GAPDH (Cell Signaling Technology).

### 2.8 Cell colony assay

Cell proliferation in CC was evaluated by plating cells at a density of 100 cells per well. Following a 10‐day culture period, colonies were immobilized using methanol and subjected to staining with a haematoxylin solution. The enumeration of colonies containing at least 50 cells was performed under a light microscope. To investigate cell chemoresistance, CC cells were exposed to cisplatin or carboplatin at specified concentrations for a duration of 6 h prior to seeding at a concentration of 500 cells per well. After an incubation period of 10 days, the colonies were fixed, stained, and captured through photography to facilitate subsequent analysis.

### 2.9 Transwell assay

To assess the migratory and invasive potential of CC cells, Transwell assays were employed. A cell suspension was introduced into the upper chamber of Transwell plates, which were either coated with Matrigel (BD Biosciences) for invasion assays or left non‐coated for migration assays. The lower chambers were supplemented with DMEM containing 10% FBS. Following incubation, the cells that migrated or invaded through the chambers were immobilized, stained and captured through photography using a light microscope.

### 2.10 Chromatin immunoprecipitation

The chromatin immunoprecipitation (ChIP) assay was performed using the EZ ChIP™ Chromatin Immunoprecipitation Kit (Millipore). HCC cells were first fixed with 1% formaldehyde and then subjected to sonication to generate chromatin fragments. Chromatin immunoprecipitation was carried out using an anti‐USF1 antibody (Abcam) and an IgG antibody (Abcam) as a control. The immunoprecipitated DNA was subsequently extracted for PCR analysis.

### 2.11 Luciferase reporter gene assay

The wild‐type PRDM4 promoter region was amplified using PCR and subsequently inserted into the pGL3‐basic dual‐luciferase reporter plasmid from Promega. This resulted in the generation of the pGL3‐PRDM4 reporter construct. The CC cells were transfected with this reporter construct. The cells were seeded in 96‐well plates at a density of 1.5 × 10^4^ cells per well. The Attractene Transfection Reagent (catalogue number 301005, QIAGEN) was utilized for the transfection process. The activity of firefly luciferase was measured and normalized by using Renilla luciferase as an internal control.

### 2.12 Statistical analysis

Statistical analysis was conducted using the SPSS software (SPSS). The two‐tailed Student's t‐test was utilized for comparing two independent groups. For comparisons involving three or more groups, one‐way anova was employed. A *p* < 0.05 was considered statistically significant.

## 3 RESULTS

### 3.1 Upregulation of circPRDM4 in chemoresistant CC cells

**FIGURE 1:**
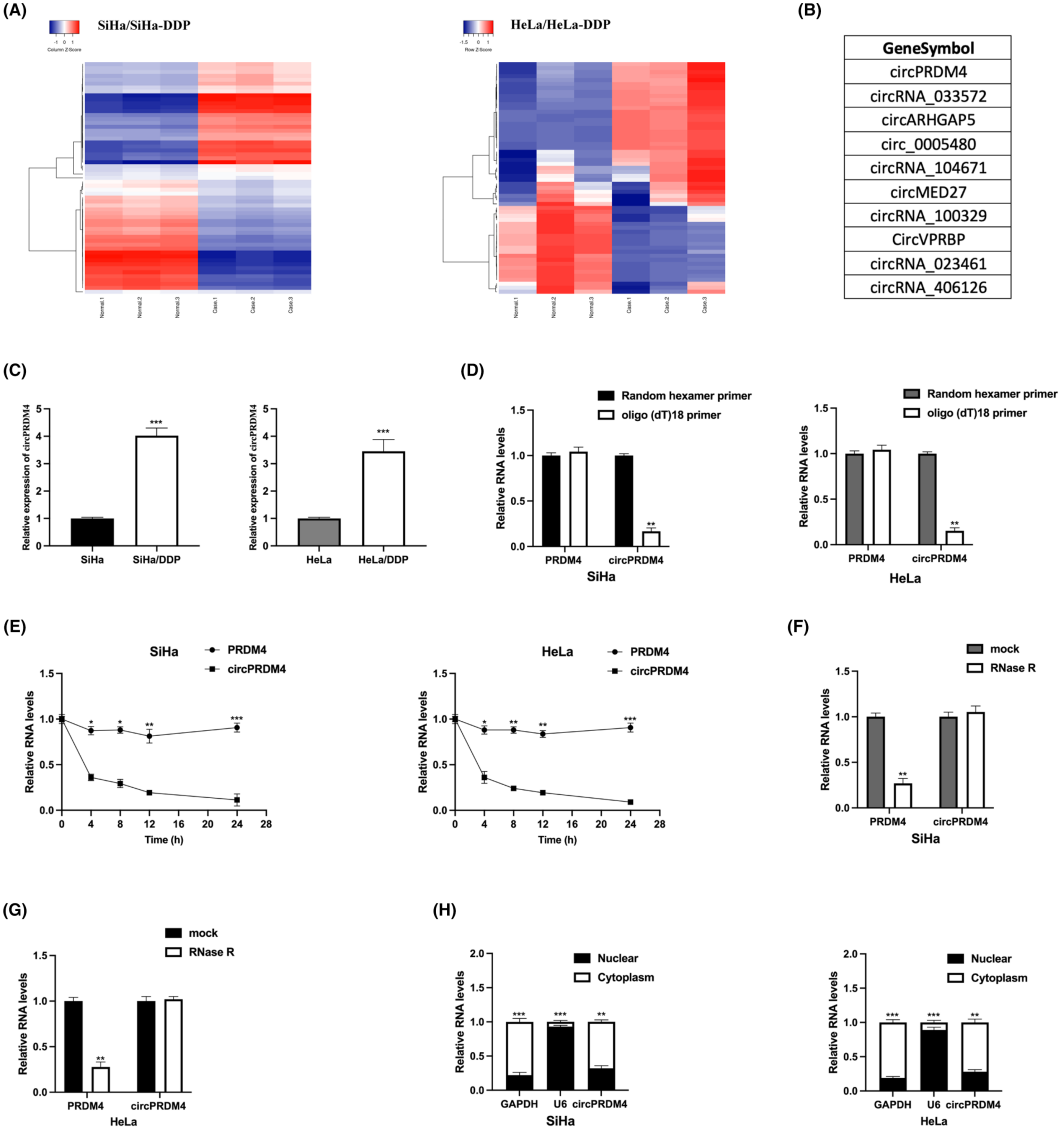
Upregulation of circPRDM4 in chemoresistant cervical cancer (CC) cells. (A) circRNA microarray analysis utilized to reveal the circRNA expression profile in DDP‐resistant CC cells. (B) The top significantly upregulated circRNAs in both DDP‐resistant CC cells. (C) CircPRDM4 expression in SiHa/SiHa‐DDP and HeLa/HeLa‐DDP cells was measured by qRT‐PCR. (D) Reverse transcription assays were conducted using either random hexamer or oligo (dT)18 primers. (E) RT‐PCR was performed to measure the relative RNA levels after treatment with actinomycin D at the specified time points. (F, G) RT‐qPCR was utilized to examine the relative RNA levels after treatment with RNase R or a mock treatment. (H) The expression of circPRDM4 in the nucleus and cytoplasm was analysed, and the results are presented as mean ± SD. All experiments were repeated three times. *p < 0.05, **p < 0.05, ***p < 0.001.

To have a better understanding of chemoresistance‐related circRNA in CC progression, circRNA microarray analysis was explored to investigate the circRNA profile of chemoresistant SiHa and HeLa to cisplatin (Figure 1A), the top 10 different expressed circRNAs in two pairs of cell lines are shown (Figure 1B), where circPRDM4 is the most significantly upregulated circRNA. A markedly upregulation of circPRDM4 in DDP‐chemoresistant SiHa and HeLa cells had been observed (Figure 1C). A noticeable decrease of circPRDM4 expression upon treatment with oligo (dT)18 primers, indicating the absence of a poly‐A tail in its structure, treatment with random hexamer primers did not affect circPRDM4 expression level (Figure 1D). The transcription inhibitor actinomycin D treatment illustrated that circPRDM4 displayed a longer half‐life than PRDM4 mRNA (Figure 1E). Furthermore, circPRDM4 exhibited significant resistance to RNase R digestion when compared to PRDM4 mRNA, as depicted in Figure 1F,G. Results of cellular RNA fractionation indicated that circPRDM4 was abundantly distributed in the cytoplasm of SiHa and HeLa cells (Figure 1H).

### 3.2 CircPRDM4 knockdown blocked immune escape of chemoresistant CC cells

**FIGURE 2:**
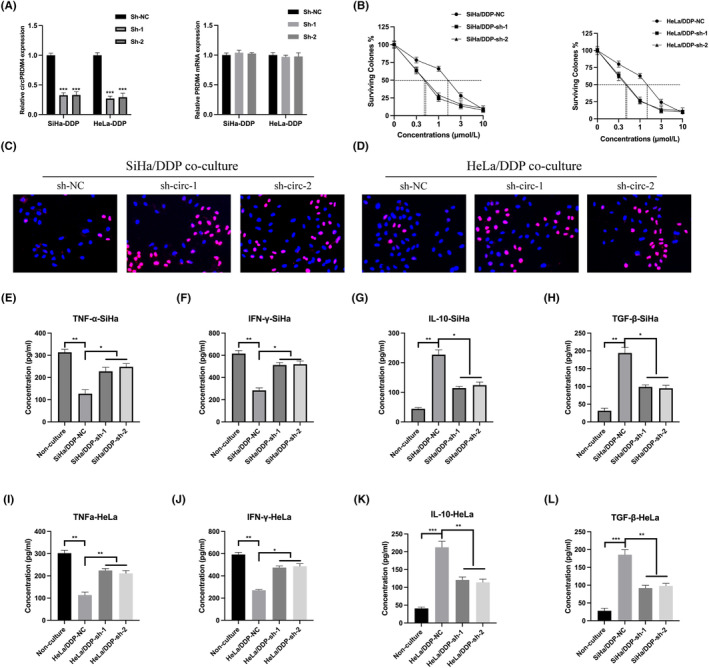
CircPRDM4 knockdown blocked immune escape of chemoresistant cervical cancer (CC) cells. (A) CircPRDM4 knockdown cell models were generated by transfecting sh‐NC, sh‐circ‐1, and sh‐circ‐2 into SiHa/SiHa‐DDP and HeLa/HeLa‐DDP cells, circPRDM4 and PRDM4 mRNA expression were detected by qRT‐PCR. (B) Cell viability were detected by CCK‐8. (C, D) Ki‐67 staining was used to measure the proliferation level of peripheral blood mononuclear cells (PBMCs) after co‐cultured with DDP‐resistant CC cells. (E–H) Enzyme‐linked immunosorbent assay (ELISA) was used to measure the expression of TNF‐α, IFN‐γ, IL‐10, and TGF‐β in the supernatant of PBMCs co‐cultured with DDP‐resistant SiHa cells. (I–L) ELISA assay was used to measure the expression of TNF‐α, IFN‐γ, IL‐10, and TGF‐β in the supernatant of PBMCs co‐cultured with DDP‐resistant HeLa cells. The results are presented as mean ± SD. All experiments were repeated three times. *p < 0.05, **p < 0.05, ***p < 0.001.

To explore the effect of circPRDM4 on CC chemoresistance, circPRDM4 knockdown models were constructed using DDP‐resistant SiHa and HeLa cells (Figure 2A). the viability of DDP‐resistant SiHa and HeLa cells treated with circPRDM4 shRNAs were detected, circPRDM4 knockdown resulted in a lower IC_50_ value compared with normal control group (Figure 2B). CircPRDM4 has been reported that modulates immune escape phenomena.^16^ Hence, DDP‐resistant SiHa and HeLa cells were used to co‐culture with PBMCs, the proliferation level of PBMCs were significantly promoted (Figure 2C). Furthermore, the expression of TNF‐α, IFN‐γ, IL‐10 and TGF‐β in the supernatant were also detected. The expression of TNF‐α and IFN‐γ were significantly reduced in the supernatant from PBMCs co‐cultured with DDP‐resistant SiHa and HeLa cells compared with non‐co‐cultured PBMCs, while these phenomena were reversed by circPRDM4 knockdown (Figure 2E,F,I,J). Moreover, IL‐10 and TGF‐β expression were markedly increased in the supernatant from PBMCs co‐cultured with DDP‐resistant SiHa and HeLa cells but reduced upon circPRDM4 knockdown (Figure 2G,H,K,L).

### 3.3 CircPRDM4 knockdown suppressed tumorigenesis of CC cells

**FIGURE 3:**
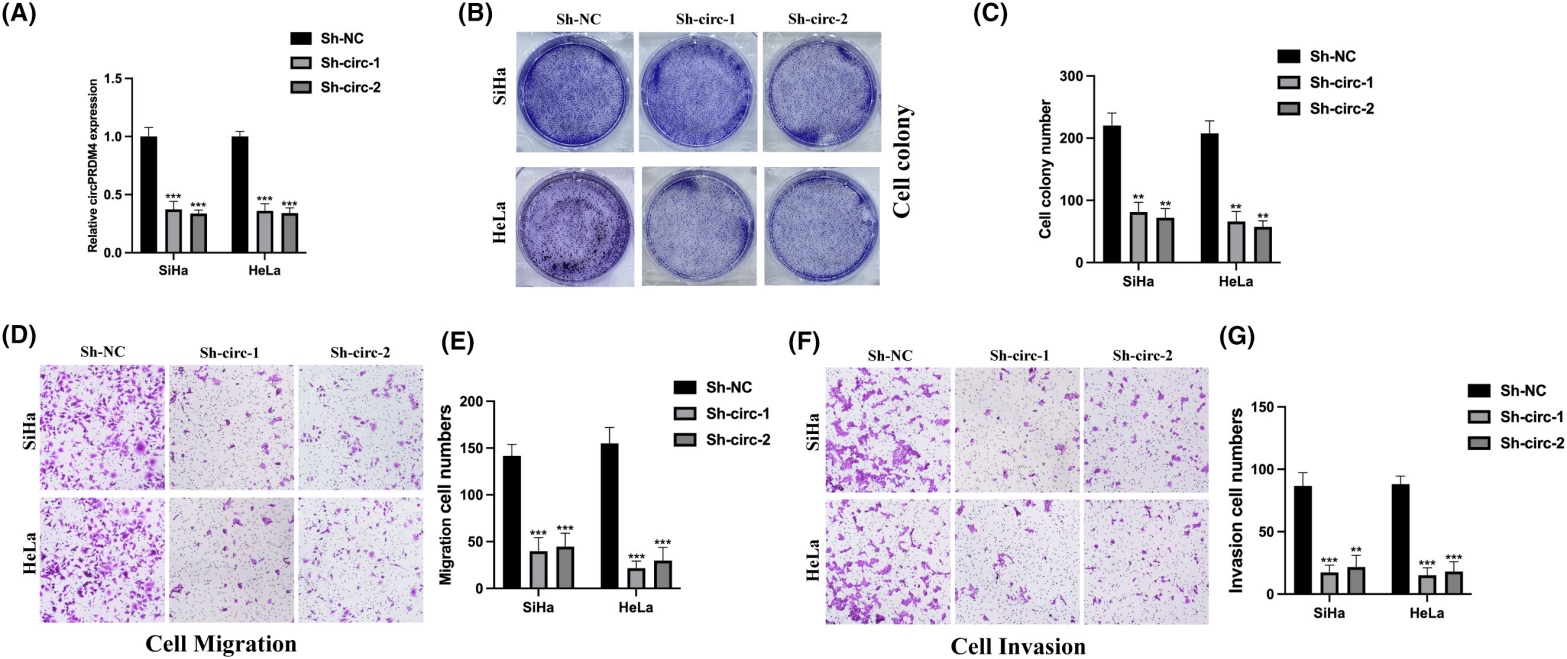
CircPDM4 knockdown suppressed tumorigenesis of cervical cancer (CC) cells. (A) CircPRMD4 knockdown cell models were constructed by stably transfecting sh‐NC, sh‐circ‐1, and sh‐circ‐2 into SiHa and HeLa cells, results were verified by qRT‐PCR. (B, C) The proliferation level of CC cells was detected by cell colony assay. (D, E) The migration level was measured by Transwell migration assay. (F, G) The invasion level was detected by Transwell invasion assay. The results are presented as mean ± SD. All experiments were repeated three times. **p < 0.05, ***p < 0.001.

In addition, circPRDM4 knockdown cell models were also generated using normal SiHa and HeLa cells to explore the biological functions of circPRDM4 in CC (Figure 3A). Cell colony results suggested that circPRDM4 knockdown significantly decreased the proliferation ability of CC cells (Figure 3B,C). Furthermore, Transwell assays were also utilized to determine cell migration and invasion ability. It was observed that circPRDM4 knockdown markedly inhibited cell migration (Figure 3D,E) and invasion (Figure 3F,G) level.

### 3.4 Transcriptional factor USF1 induced circPRDM4 expression

**FIGURE 4:**
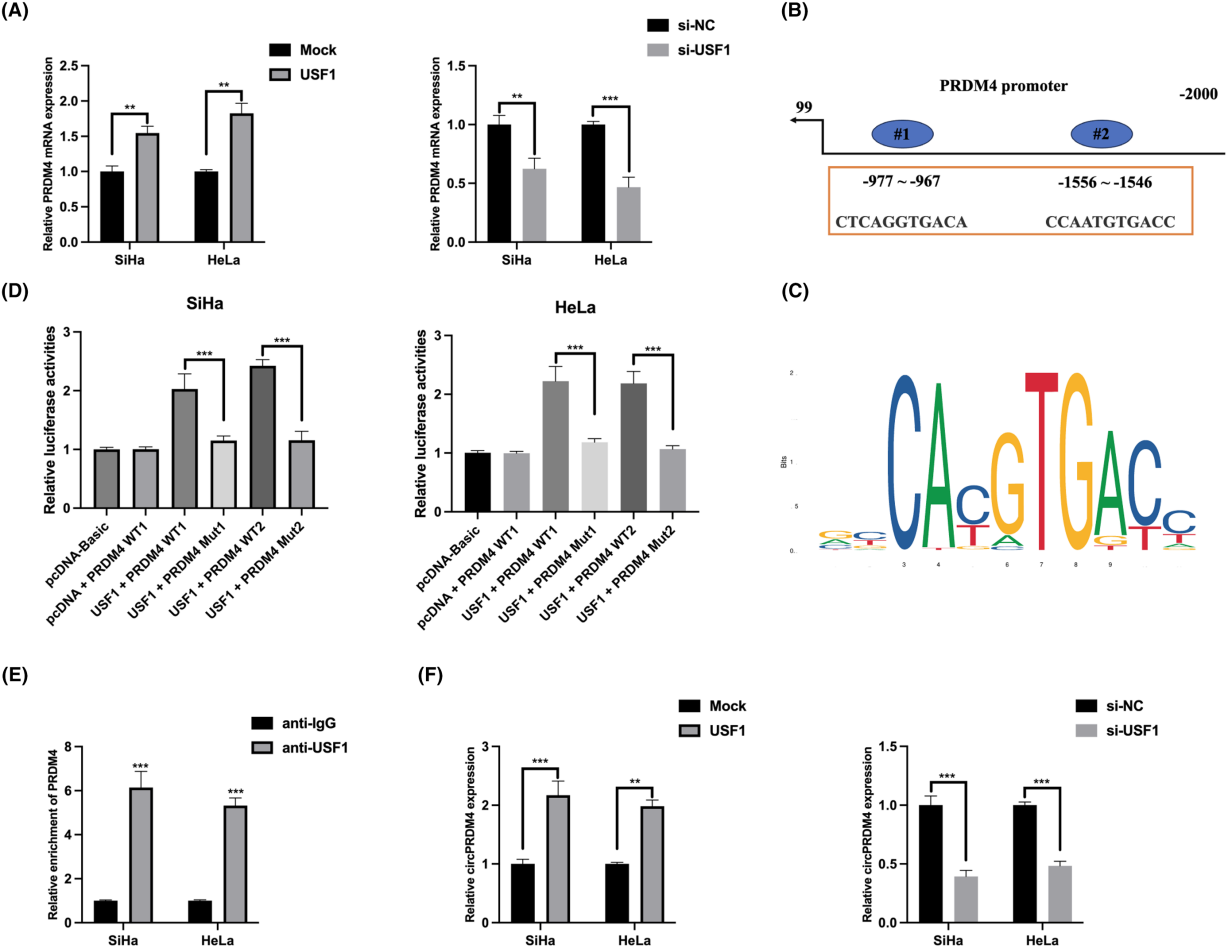
Transcriptional factor USF1 induced circPRDM4 expression. (A) Relative expression of PRDM4 mRNA in USF1 overexpression or knockdown cell models were detected by qRT‐PCR. (B, C) The graphical illustration of the binding sites between USF1 and PRDM4 promoter. (D) Luciferase reporter gene assay was performed to evaluate the binding fact between USF1 and PRDM4 promoter. (E) ChIP assay was utilized using anti‐IgG and anti‐USF1, results were detected by qRT‐PCR. (F) CircPRDM4 expression in USF1 overexpression or knockdown cell models were detected by qRT‐PCR. The results are presented as mean ± SD. All experiments were repeated three times. **p < 0.05, ***p < 0.001.

Previous studies have well demonstrated that transcription factor plays essential role in circRNA expression dysregulation.^17^, ^18^, ^19^ To reveal the upregulation of circPRDM4 in CC, JASPAR database was utilized, USF1 was found as a potential transcriptional factor of circPRDM4. Next, it was found that PRDM4 mRNA expression could be positively regulated by USF1 in CC cells (Figure 4A). USF1 has two binding sites on the upstream region of PRDM4 mRNA transcript (Figure 4B) and the binding sequence of USF1 was also obtained (Figure 4C). Luciferase reporter gene assay elucidated that USF1 enhanced the luciferase activity of USF1 + PRDM4 WT1/2, but not USF1 + PRDM4 Mut1/2, indicating that USF1 bound to PRDM4 promoter two regions (Figure 4D). ChIP assay showed that PRDM4 significantly enriched by anti‐USF1 (Figure 4E). As indicated in Figure 4F, circPRDM4 expression in CC cells could be positively regulated by USF1.

### 3.5 USF1 participated in the progression of CC cell immune escape via circPRDM4

**FIGURE 5:**
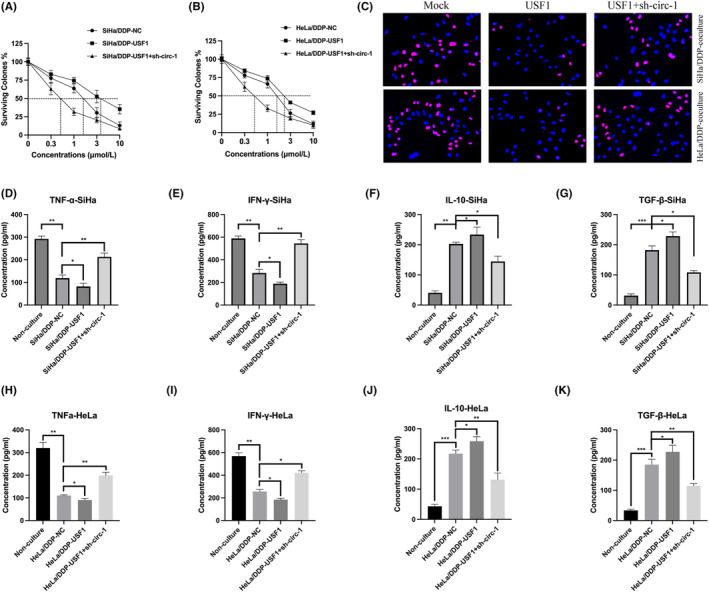
USF1 blocked cervical cancer (CC) cell immune escape via circPRDM4. (A, B) Cell viability were detected by CCK‐8. (C) Ki‐67 staining was used to measure the proliferation level of peripheral blood mononuclear cells (PBMCs) after co‐cultured with DDP‐resistant CC cells. (D–G) Enzyme‐linked immunosorbent assay (ELISA) was used to measure the expression of TNF‐α, IFN‐γ, IL‐10 and TGF‐β in the supernatant of PBMCs co‐cultured with DDP‐resistant SiHa cells. (H–K) ELISA assay was used to measure the expression of TNF‐α, IFN‐γ, IL‐10, and TGF‐β in the supernatant of PBMCs co‐cultured with DDP‐resistant HeLa cells. The results are presented as mean ± SD. All experiments were repeated three times. *p < 0.05, **p < 0.05, ***p < 0.001.

To elucidate the USF1/circPRDM4 axis in CC chemoresistance, DDP‐resistant CC cell models were generated through stably transfecting USF1 and sh‐circ‐1. USF1 overexpression resulted in a higher IC50 value compared with normal control group, but this effect reversed by circPRDM4 knockdown (Figure 5A,B). Regarding to the immune escape detection, PBMC proliferation ability was markedly inhibited by USF1 overexpression but following been promoted by circPRDM4 knockdown (Figure 5C). Subsequently, the expression of TNF‐α and IFN‐γ were significantly induced in the supernatant from PBMCs co‐cultured with USF1 overexpression DDP‐resistant cells and were inhibited by circPRDM4 knockdown (Figure 5D,E,H,I). IL‐10 and TGF‐β expression were markedly increased but reduced upon circPRDM4 knockdown (Figure 5F,G,J,K).

### 3.6 USF1 involved in the tumorigenesis of CC cells through regulating circPRDM4

**FIGURE 6:**
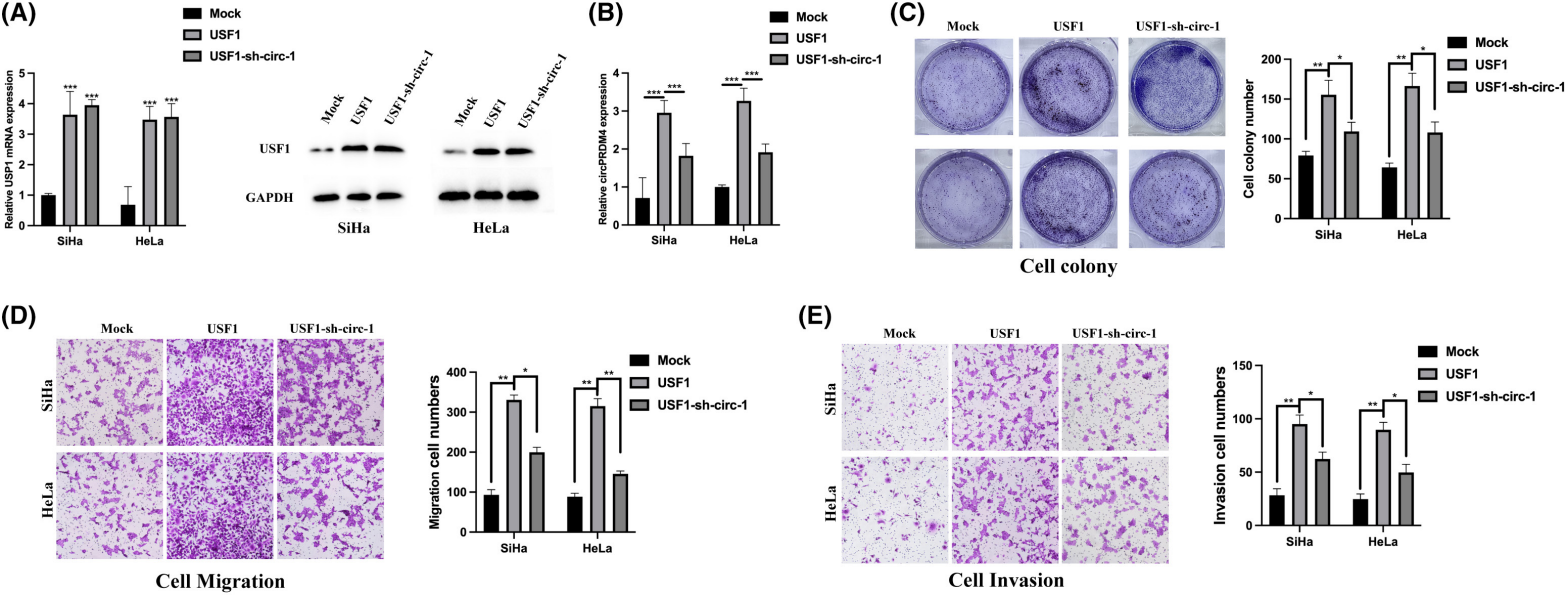
USF1 involved in the tumorigenesis of cervical cancer cells through regulating circPRDM4. (A) USF1 expression was detected by qRT‐PCR and western blot. (B) CircPRDM4 expression was measured by qRT‐PCR. (C) Cell colony assay was performed to evaluate cell proliferation level. (D) Transwell assay was conducted to detect cell migration level. (E) Cell invasion level was assessed by Transwell assay. The results are presented as mean ± SD. All experiments were repeated three times. *p < 0.05, **p < 0.05, ***p < 0.001.

The tumorigenesis functions of USF1/circPRDM4 axis were also explored. The expression of USF1 and circPRDM1 were detected by western blot and qRT‐PCR (Figure 6A,B). The proliferation level of CC cells was significantly promoted by USF1 overexpression and attenuated by circPRDM4 knockdown (Figure 6C). The same phenomena were also observed in Transwell migration (Figure 6E) and Transwell invasion (Figure 6E) assays. Those findings suggested that USF1 modulates immune escape and tumorigenesis in chemoresistant CC cells.

## 4 DISCUSSION

Cisplatin is regarded as the most efficacious drug for neoadjuvant and salvage treatment of CC.^20^ However, the therapeutic efficacy of cisplatin‐based chemotherapy is significantly compromised by the inherent resistance to cisplatin as well as the development of acquired resistance.^1^, ^5^ The intricate mechanism responsible for the inherent insensitivity and acquired resistance to cisplatin is complex and not yet fully comprehended. Hence, uncover the underlying mechanisms of chemoresistance of CC in‐depth is urgently needed. By conducting circRNA microarray analysis on two DDP‐chemoresistant CC cell lines, circPRDM4 was identified as a most significantly upregulated circRNA. CircPRDM4 knockdown markedly re‐sensitive CC cells to cisplatin treatment and blocked immune escape of CC cells.

In the past few years, significant emphasis has been placed on understanding the impact of immune evasion in the development of CC. Cellular immunity, particularly involving T lymphocytes, dendritic cells, natural killer cells and macrophages, plays a crucial role in anti‐tumour immune responses. Nevertheless, tumours can persist and metastasize despite the presence of a functioning immune system, suggesting that tumours possess mechanisms to evade immune surveillance.^21^, ^22^, ^23^ Previous study has demonstrated that circPRDM4 participates in immune escape in hepatocellular carcinoma.^16^ Thereafter, the role of circPRDM4 in the immune response of DDP‐resistant CC cells was also explored for a better understanding of the phenomena of circPRDM4 on CC chemoresistance. CircPRDM4 knockdown markedly blocked CC cell immune escape when co‐cultured with PBMCs.

Subsequently, to uncover underlying mechanisms of circPRDM4 upregulation upon DDP treatment, the upstream regulator of circPRDM4 was investigated. Many studies have reported that transcription factor could induce circRNA expression. The transcription factor Twist1 has been found to enhance the transcription of circ‐Cul2 by binding to its promoter.^24^ Additionally, E2F1 and EIF4A3 have been identified as factors that can elevate the expression level of circSEPT9.^25^ Another transcription factor, Sp1, characterized by its zinc finger structure, binds to GC‐rich DNA motifs and plays a regulatory role in numerous genes. Sp1 is involved in essential cellular processes including cell growth, differentiation and apoptosis.^26^, ^27^ In this study, USF1 was identified as a transcription factor of circPRDM4 and induced circPRDM4 upregulation in CC cells. USF1 and USF2, members of the basic helix–loop–helix leucine zipper family, are classified as USFs.^28^ USF1, in particular, serves as a crucial regulatory factor and exhibits ubiquitous expression across various tissues. It predominantly binds to the consensus sequence (CANNTG) of E‐box and subsequently exerts transcriptional control over different genes.^29^ Previous research has demonstrated that aberrant expression of USF1 can initiate tumour formation and facilitate the progression of tumours.^30^, ^31^, ^32^, ^33^ While the understanding of USF1 in chemoresistant CC is limited. Our study elucidated that USF1 modulated immune escape of DDP‐resistant CC cells and tumorigenesis through upregulating circPRDM4.

In summary, this study partially elucidated the USF1/circPRDM4 axis in the progression of immune escape of DDP‐resistant CC cells and tumorigenesis, which might provide valuable insights into the mechanisms underlying immune escape and tumorigenesis.

## AUTHOR CONTRIBUTIONS

**Yan Zhang:** Conceptualization (equal); investigation (equal); project administration (equal); supervision (equal); writing – original draft (equal). **Xing Li:** Data curation (equal); formal analysis (equal); methodology (equal); software (equal). **Jun Zhang:** Data curation (equal); methodology (equal); resources (equal); validation (equal). **Lin Mao:** Formal analysis (equal); resources (equal); software (equal); visualization (equal). **Zou Wen:** Formal analysis (equal); software (equal); visualization (equal). **Mingliang Cao:** Formal analysis (equal); software (equal); validation (equal). **Xuefeng Mu:** Data curation (equal); methodology (equal); validation (equal).

## CONFLICT OF INTEREST STATEMENT

The authors declare that they have no competing interests.

## Data Availability

The data supporting this study's findings are available from the corresponding author upon reasonable request. Some data may not be made available because of privacy or ethical restrictions.

## References

[1] Masadah R , Rauf S , Pratama MY , Tiribelli C , Pascut D . The role of microRNAs in the cisplatin‐ and radio‐resistance of cervical cancer. Cancers (Basel). 2021;13(5):1168. doi:10.3390/cancers13051168 33803151 PMC7963155

[2] Diaz‐Padilla I , Monk BJ , Mackay HJ , Oaknin A . Treatment of metastatic cervical cancer: future directions involving targeted agents. Crit Rev Oncol Hematol. 2013;85(3):303‐314. doi:10.1016/j.critrevonc.2012.07.006 22883215

[3] Weingart SN , Zhang L , Sweeney M , Hassett M . Chemotherapy medication errors. Lancet Oncol. 2018;19(4):e191‐e199. doi:10.1016/S1470-2045(18)30094-9 29611527

[4] Morrow M , Khan AJ . Locoregional management after Neoadjuvant chemotherapy. J Clin Oncol. 2020;38(20):2281‐2289. doi:10.1200/JCO.19.02576 32442069 PMC7343435

[5] Bhattacharjee R , Dey T , Kumar L , et al. Cellular landscaping of cisplatin resistance in cervical cancer. Biomed Pharmacother. 2022;153:113345. doi:10.1016/j.biopha.2022.113345 35810692

[6] Zhou WY , Cai ZR , Liu J , Wang DS , Ju HQ , Xu RH . Circular RNA: metabolism, functions and interactions with proteins. Mol Cancer. 2020;19(1):172. doi:10.1186/s12943-020-01286-3 33317550 PMC7734744

[7] Liu CX , Chen LL . Circular RNAs: characterization, cellular roles, and applications. Cell. 2022;185(12):2016‐2034. doi:10.1016/j.cell.2022.04.021 35584701

[8] Kristensen LS , Andersen MS , Stagsted LVW , Ebbesen KK , Hansen TB , Kjems J . The biogenesis, biology and characterization of circular RNAs. Nat Rev Genet. 2019;20(11):675‐691. doi:10.1038/s41576-019-0158-7 31395983

[9] Patop IL , Wust S , Kadener S . Past, present, and future of circRNAs. EMBO J. 2019;38(16):e100836. doi:10.15252/embj.2018100836 31343080 PMC6694216

[10] Chen L , Shan G . CircRNA in cancer: fundamental mechanism and clinical potential. Cancer Lett. 2021;505:49‐57. doi:10.1016/j.canlet.2021.02.004 33609610

[11] Wu P , Mo Y , Peng M , et al. Emerging role of tumor‐related functional peptides encoded by lncRNA and circRNA. Mol Cancer. 2020;19(1):22. doi:10.1186/s12943-020-1147-3 32019587 PMC6998289

[12] Kristensen LS , Jakobsen T , Hager H , Kjems J . The emerging roles of circRNAs in cancer and oncology. Nat Rev Clin Oncol. 2022;19(3):188‐206. doi:10.1038/s41571-021-00585-y 34912049

[13] Chen LL . The expanding regulatory mechanisms and cellular functions of circular RNAs. Nat Rev Mol Cell Biol. 2020;21(8):475‐490. doi:10.1038/s41580-020-0243-y 32366901

[14] Chen M , Ai G , Zhou J , Mao W , Li H , Guo J . circMTO1 promotes tumorigenesis and chemoresistance of cervical cancer via regulating miR‐6893. Biomed Pharmacother. 2019;117:109064. doi:10.1016/j.biopha.2019.109064 31226633

[15] Guo J , Chen M , Ai G , Mao W , Li H , Zhou J . Hsa_circ_0023404 enhances cervical cancer metastasis and chemoresistance through VEGFA and autophagy signaling by sponging miR‐5047. Biomed Pharmacother. 2019;115:108957. doi:10.1016/j.biopha.2019.108957 31082770

[16] Chen ZQ , Zuo XL , Cai J , et al. Hypoxia‐associated circPRDM4 promotes immune escape via HIF‐1α regulation of PD‐L1 in hepatocellular carcinoma. Exp Hematol Oncol. 2023;12(1):17. doi:10.1186/s40164-023-00378-2 36747292 PMC9903500

[17] Ren L , Jiang M , Xue D , et al. Nitroxoline suppresses metastasis in bladder cancer via EGR1/circNDRG1/miR‐520h/smad7/EMT signaling pathway. Int J Biol Sci. 2022;18(13):5207‐5220. doi:10.7150/ijbs.69373 35982887 PMC9379395

[18] Jiang Y , Zhou J , Zhao J , et al. The U2AF2 /circRNA ARF1/miR‐342‐3p/ISL2 feedback loop regulates angiogenesis in glioma stem cells. J Exp Clin Cancer Res. 2020;39(1):182. doi:10.1186/s13046-020-01691-y 32894165 PMC7487667

[19] Jiang Z , Tai Q , Xie X , et al. EIF4A3‐induced circ_0084615 contributes to the progression of colorectal cancer via miR‐599/ONECUT2 pathway. J Exp Clin Cancer Res. 2021;40(1):227. doi:10.1186/s13046-021-02029-y 34253241 PMC8273970

[20] Miriyala R , Mahantshetty U , Maheshwari A , Gupta S . Neoadjuvant chemotherapy followed by surgery in cervical cancer: past, present and future. Int J Gynecol Cancer. 2022;32(3):260‐265. doi:10.1136/ijgc-2021-002531 35256411

[21] Aktar N , Yueting C , Abbas M , et al. Understanding of immune escape mechanisms and advances in cancer immunotherapy. J Oncol. 2022;2022:8901326. doi:10.1155/2022/8901326 35401745 PMC8989557

[22] Chen F , Shen L , Wang Y , et al. Signatures of immune cell infiltration for predicting immune escape and immunotherapy in cervical cancer. Aging (Albany NY). 2023;15(5):1685‐1698. doi:10.18632/aging.204583 36917087 PMC10042703

[23] Pansy K , Uhl B , Krstic J , et al. Immune regulatory processes of the tumor microenvironment under malignant conditions. Int J Mol Sci. 2021;22(24). doi:10.3390/ijms222413311 PMC8706102 34948104

[24] Meng J , Chen S , Han JX , et al. Twist1 regulates Vimentin through Cul2 circular RNA to promote EMT in hepatocellular carcinoma. Cancer Res. 2018;78(15):4150‐4162. doi:10.1158/0008-5472.CAN-17-3009 29844124

[25] Zheng X , Huang M , Xing L , et al. The circRNA circSEPT9 mediated by E2F1 and EIF4A3 facilitates the carcinogenesis and development of triple‐negative breast cancer. Mol Cancer. 2020;19(1):73. doi:10.1186/s12943-020-01183-9 32264877 PMC7137343

[26] Nylén C , Aoi W , Abdelmoez AM , et al. IL6 and LIF mRNA expression in skeletal muscle is regulated by AMPK and the transcription factors NFYC, ZBTB14, and SP1. Am J Physiol Endocrinol Metab. 2018;315(5):E995‐E1004. doi:10.1152/ajpendo.00398.2017 29688769

[27] Tan NY , Khachigian LM . Sp1 phosphorylation and its regulation of gene transcription. Mol Cell Biol. 2009;29(10):2483‐2488. doi:10.1128/MCB.01828-08 19273606 PMC2682032

[28] Sa L , Li Y , Zhao L , et al. The role of HOTAIR/miR‐148b‐3p/USF1 on regulating the permeability of BTB. Front Mol Neurosci. 2017;10:194. doi:10.3389/fnmol.2017.00194 28701916 PMC5487514

[29] Wu H , Qiao M , Peng X , et al. Molecular characterization, expression patterns, and association analysis with carcass traits of porcine USF1 gene. Appl Biochem Biotechnol. 2013;170(6):1310‐1319. doi:10.1007/s12010-013-0280-5 23666615

[30] Sun Q , Li J , Li F , et al. LncRNA LOXL1‐AS1 facilitates the tumorigenesis and stemness of gastric carcinoma via regulation of miR‐708‐5p/USF1 pathway. Cell Prolif. 2019;52(6):e12687. doi:10.1111/cpr.12687 31468594 PMC6869681

[31] Ren YQ , Li QH , Liu LB . USF1 prompt melanoma through upregulating TGF‐β signaling pathway. Eur Rev Med Pharmacol Sci. 2016;20(17):3592‐3598. 27649659

[32] Zeng K , He B , Yang BB , et al. The pro‐metastasis effect of circANKS1B in breast cancer. Mol Cancer. 2018;17(1):160. doi:10.1186/s12943-018-0914-x 30454010 PMC6240936

[33] Li P , Wang H , Hou M , Li D , Bai H . Upstream stimulating factor1 (USF1) enhances the proliferation of glioblastoma stem cells mainly by activating the transcription of mucin13 (MUC13). Pharmazie. 2017;72(2):98‐102. doi:10.1691/ph.2017.6788 29441861

